# Data on microbial assessment and physicochemical characteristics of sachet water samples obtained from three factories in Ota, Ogun state, Nigeria

**DOI:** 10.1016/j.dib.2018.07.035

**Published:** 2018-07-21

**Authors:** Oluwaseun J. Okunola, Deborah O. Oba, Solomon U. Oranusi, Hilary I. Okagbue

**Affiliations:** a Department of Biological Sciences Covenant University, Ota, Nigeria; b Department of Mathematics Covenant University, Ota, Nigeria

**Keywords:** Satchet water, Microbial diversity, Water, Physiochemical, Coliform, Statistics

## Abstract

The data described in this article were obtained in a study to assess the bacteriological and physicochemical properties of packaged sachet water sold for public consumption. Sixty sachet water samples from 3 different brands (A, B and C) produced and consumed in Ota, Ogun State was collected. Stratified sampling method was used. Samples were subjected to microbiological analysis using pour plate method and colony counter to count the organisms. The packaged water samples were assessed for the total coliform count, total plate count and count on Salmonella- Shigella agar. Physicochemical parameters were also assayed for and reported here. The analysis of the data presented here can be helpful in improving public health and creating awareness of the risk of consumption of satchel water.

**Specifications Table**

**Table:** 

Subject area	*Microbiology*
More specific subject area	*Public Health Microbiology*
Type of data	*Tables, figures*
How data was acquired	*Colony counter(Stuart, serial R000102178), photometer(Model 7500 PHOT.1.1.AUTO.75)*
Data format	*Raw*
Experimental factors	*Microbial counts, physicochemical parameters measurement*
Experimental features	Sachet water samples were obtained from different manufacturers. The packaged water samples were assessed for total coliform count, total plate count and count on Salmonella- Shigella agar. The physicochemical properties of water samples were also assessed.
Data source location	*Ota, Ogun State, Nigeria.*
Data accessibility	*Data available within the article*

**Value of the data**

• The data presented could provide a holistic assessment of water standards in Nigeria when merged with data from other regions.
• Data presented in this article could inform intervention programs targeted towards improving drinking water condition in the region under review.
• Data presented here can influence policies on water distribution in the region under review.
• The data can be useful for regulatory agencies and in auditing water quality as stated in the Sustainable Development Goals (SDG).

## 1 Data

The data were obtained in a study to assess the bacteriological and physicochemical properties of packaged sachet water sold for public consumption. Sixty sachet water samples from 3 different brands (A, B and C) produced and consumed in Ota, Ogun State were collected.Samples were subjected to microbiological analysis using pour plate method and colony counter to count the organisms. The packaged water samples were assessed for the following: a). total coliform count, b). total plate count and c). count on Salmonella- Shigella agar. These are presented in Table 1, Table 2, Table 3 along with their respective descriptive statistics.

**Table 1:** Microbial count of sachet water from brand A.

Samples	Total plate count (Cfu/ml)	Count on *Salmonella- Shigella* agar (Cfu/ml)	Total Coliform count (Cfu/ml)
NIS	–	–	10
WHO Standard	–	–	10
A1	1.6 × 10^2^	3 × 10^1^	5 × 10^1^
A2	9 × 10^1^	NG	NG
A3	1.2 × 10^2^	2 × 10^1^	4 × 10^1^
A4	1.7 × 10^2^	NG	1 × 10^1^
A5	1.1 × 10^2^	1 × 10^1^	NG
A6	1.6 × 10^2^	3 × 10^1^	5 × 10^1^
A7	9 × 10^1^	NG	1 × 10^1^
A8	1.5 × 10^2^	NG	NG
A9	8 × 10^1^	1 × 10^1^	NG
A10	1.3 × 10^2^	2 × 10^1^	NG
A11	1.3 × 10^2^	NG	3 × 10^1^
A12	6 × 10^1^	NG	2 × 10^1^
A13	1 × 10^1^	NG	NG
A14	1.2 × 10^2^	4 × 10^1^	2 × 10^1^
A15	5 × 10^1^	NG	NG
A16	9 × 10^1^	3 × 10^1^	4 × 10^1^
A17	1.1 × 10^2^	NG	3 × 10^1^
A18	NG	NG	NG
A19	9 × 10^1^	4 × 10^1^	2 × 10^1^
A20	1.5 × 10^2^	NG	5 × 10^1^
Mean	1.0 × 10^2^	1.1 × 10^1^	1.8 × 10^1^
Standard Deviation	4.7 × 10^1^	1.5 × 10^1^	1.9 × 10^1^

NG: No growth, NIS: Nigeria Industrial Standards, WHO: World Health Organizations.

**Table 2:** Microbial count of sachet water from brand B.

Samples	Total plate count (Cfu/ml)	Count on *Salmonella- Shigella* agar (Cfu/ml)	Total Coliform count (Cfu/ml)
NIS	–	–	10
WHO Standard	–	–	10
B1	3 × 10^1^	NG	NG
B2	1.4 × 10^2^	4 × 10^1^	4 × 10^1^
B3	NG	NG	NG
B4	6 × 10^1^	1 × 10^1^	NG
B5	1.2 × 10^2^	3 × 10^1^	5 × 10^1^
B6	8 × 10^1^	NG	3 × 10^1^
B7	4 × 10^1^	NG	NG
B8	1.3 × 10^2^	2 × 10^1^	3 × 10^1^
B9	4 × 10^1^	NG	2 × 10^1^
B10	7 × 10^1^	NG	NG
B11	9 × 10^1^	NG	2 × 10^1^
B12	4 × 10^1^	NG	3 × 10^1^
B13	7 × 10^1^	3 × 10^1^	NG
B14	NG	NG	NG
B15	5 × 10^1^	NG	2 × 10^1^
B16	3 × 10^1^	2 × 10^1^	NG
B17	1 × 10^2^	3 × 10^1^	2 × 10^1^
B18	8 × 10^1^	NG	3 × 10^1^
B19	NG	NG	NG
B20	4 × 10^1^	2 × 10^1^	3 × 10^1^
Mean	6 × 10^1^	1 × 10^1^	1.6 × 10^1^
Standard deviation	4.1 × 10^1^	1.4 × 10^1^	1.6 × 10^1^

NG: No growth, NIS: Nigeria Industrial Standards, WHO: World Health Organizations.

**Table 3:** Microbial count of sachet water from brand C.

Samples	Total plate count (Cfu/ml)	Count on *Shigella salmonella* agar (Cfu/ml)	Total Coliform count (Cfu/ml)
NIS	–	–	10
WHO Standard	–	–	10
C1	NG	2 × 10^1^	NG
C2	8 × 10^1^	NG	1 × 10^1^
C3	3 × 10^1^	NG	NG
C4	7 × 10^1^	NG	4 × 10^1^
C5	NG	NG	NG
C6	2 × 10^1^	NG	1 × 10^1^
C7	4 × 10^1^	3 × 10^1^	NG
C8	1 × 10^1^	NG	1 × 10^1^
C9	6 × 10^1^	2 × 10^1^	NG
C10	9 × 10^1^	2 × 10^1^	NG
C11	4 × 10^1^	NG	3 × 10^1^
C12	6 × 10^1^	1 × 10^1^	4 × 10^1^
C13	8 × 10^1^	NG	NG
C14	7 × 10^1^	NG	NG
C15	8 × 10^1^	2 × 10^1^	6 × 10^1^
C16	NG	NG	NG
C17	3 × 10^1^	NG	1 × 10^1^
C18	4 × 10^1^	NG	NG
C19	7 × 10^1^	1 × 10^1^	5 × 10^1^
C20	4 × 10^1^	NG	NG
Mean	4.6 × 10^1^	1 × 10^1^	1.3 × 10^1^
Standard deviation	2.9 × 10^1^	1 × 10^1^	1.9 × 10^1^

NG: No growth, NIS: Nigeria Industrial Standards, WHO: World Health Organizations.

Statistical analysis can be performed using the raw data. In addition, public health concern of the consumption of sachet water is the main reason why the data were obtained and comparisons were made on the permitted levels of the physiochemical parameters as stated by the Nigerian Industrial Standards: Standard Organization of Nigeria (NIS) and the World Health Organization (WHO).

## 2 Experimental design, materials and methods

The experimental design in this article is similar to the ones found in [1], [2], [3], [4], [5], [6], [7], [8], [9], [10], [11], [12] However; different statistical methods can be discussed in [13], [14], [15], [16], [17], [18], [19], [20], [21], [22], [23] may be adopted in the analysis of the raw data.

### 2.1 Study area description

Sachet water samples were obtained from three factories in Ota, Ogun State, Nigeria. Stratified sampling method was used. The choice of the method is to distinctly identify and characterize the microbial content of the sachet water and to investigate whether the microbial content is the same or different between the brands.

### 2.2 Sample Collection and analytical procedure

The samples were collected from February to March, 2018. A 1/10 dilution was performed. Aliquot of the dilution was inoculated onto plates of Eosin methylene blue agar (EMB), *Salmonella-shigella* agar (SSA) and Nutrient agar (NA) using the pour plate method. Cultures were allowed to grow for 18–24 h, after which the resulting colonies were enumerated using the colony counter. Colony counts were converted to colony forming units using the formula below;

$$\mathrm{Colony\backslash forming\backslash unit}=\;\frac{\mathrm{No\backslash of\backslash Colonie}}{\mathrm{volume\backslash plated}}÷\mathrm{dilution\backslash factor\backslash cfu}/\mathrm{ml}$$

### 2.3 Physicochemical analysis

Physicochemical analysis was conducted using a photometer. Measurements were done in triplicates. This can be seen in Table 4. The reliability of the data is improved by the replication. Hence, further statistical tools can be applied.

**Table 4:** Physicochemical analysis of sachet water from brands A, B and C.

**Parameters (mg/L)**	**A**	**B**	**C**	**NIS**	**WHO**
Aluminium	ND	ND	ND	0.2	0.2
Calcium Hardness	ND	ND	ND	–	–
Chloride	0	0	0.3, 0.3, 0.5	250	250
Dissolve oxygen	0	0	0.01, 0.02.0,02	–	6
Fluoride	ND	ND	ND	1.5	1.5
Free chlorine	0	0.05,0.04, 0.06	0.06, 0.07.0.07	0.2–0.025	0.2
Iron	0	0	ND	0.3	0.3
Magnesium	ND	0	ND	0.20	0.20
Manganese	ND	0	ND	0.2	0.2
Nitrite	0	0	0	0.2	0.2
Phosphate	0	0	ND	–	3.50
Potassium	0.3, 0.3, 0.2	0	0.2, 0.2, 0.2	–	–
Sulphate	4.0, 3.8, 4.2	0	3.0, 3.1,3.1	–	0.002
Total Alkalinity(CaCO_3_)	ND	ND	ND	–	–
Total Chlorine	0	0.004, 0.03,0.02	0.05, 0.06.0.05	–	–
Total Copper	0.02, 0.03, 0.02	0	0.2, 0.1, 0.2	–	2
Total Hardness	ND	ND	ND	–	500
Total Nickel	ND	0	0.5, 0.3, 0.5	0,02	0.02
Total Phosphorus	ND	0	0.12, 0.10, 0.13	–	–
Turbidity (NTU)	4.0, 4.0, 4.3	0	4.0, 4.0, 4.2		5
Zinc	ND	ND	ND	3	–

ND: Not Detected, NIS: Nigeria Industrial Standards, WHO: World Health Organizations.

### 2.4 Analysis of variance

Some analysis of variance (ANOVA) tests was conducted to determine the extent of significance of mean differences among the variable parameters of microbial composition. These are presented in Table 5, Table 6, Table 7, Table 8, Table 9, Table 10.

**Table 5:** ANOVA of microbial count of sachet water from brand A.

*Source of variation*	*SS*	*Df*	*MS*	*F*	*P-value*	*F criteria*
Between Groups	1049.2	2	524.6	55.41036	< 0.0000	3.158843
Within Groups	539.65	57	9.467544			
Total	1588.85	59				

**Remark:** There is a significant difference among the means of the microbial count parameters of brand A.

**Table 6:** ANOVA of microbial count of sachet water from brand B.

*Source of variation*	*SS*	*Df*	*MS*	*F*	*P-value*	*F criteria*
Between Groups	304.4333	2	152.2167	20.97003	< 0.0000	3.158843
Within Groups	413.75	57	7.258772			
Total	718.1833	59				

**Remark:** There is a significant difference among the means of the microbial count parameters of brand B.

**Table 7:** ANOVA of microbial count of sachet water from brand C.

*Source of variation*	*SS*	*Df*	*MS*	*F*	*P-value*	*F criteria*
Between Groups	174.6333	2	87.31667	19.46441	<0.0000	3.158843
Within Groups	255.7	57	4.485965			
Total	430.3333	59				

**Remark:** There is a significant difference among the means of the microbial count parameters of brand C.

**Table 8:** ANOVA of Total plate count (Cfu/ml) of brands A, B and C.

*Source of variation*	*SS*	*Df*	*MS*	*F*	*P-value*	*F criteria*
Between Groups	362.5333	2	181.2667	11.24961	7.62E−05	3.158843
Within Groups	918.45	57	16.11316			
Total	1280.983	59				

**Remark:** There is a significant difference among the means of the total plate count of the three brands.

**Table 9:** ANOVA of Count on Salmonella- Shigella agar (Cfu/ml) of brands A, B and C.

*Source of variation*	*SS*	*Df*	*MS*	*F*	*P-value*	*F criteria*
Between Groups	2.633333	2	1.316667	0.772915	0.466443	3.158843
Within Groups	97.1	57	1.703509			
Total	99.73333	59				

**Remark:** There is no significant difference among the means of the count on Salmonella- Shigella agar of the three brands.

**Table 10:** ANOVA of total Coliform count (Cfu/ml) of brands A, B and C.

*Source of variation*	*SS*	*Df*	*MS*	*F*	*P-value*	*F criteria*
Between Groups	3.033333	2	1.516667	0.446655	0.641985	3.158843
Within Groups	193.55	57	3.395614			
Total	196.5833	59				

**Remark:** There is no significant difference among the means of the total Coliform count of the three brands.
